# The association of feeding difficulties and generic health-related quality of life among children born with esophageal atresia

**DOI:** 10.1186/s13023-023-02836-w

**Published:** 2023-08-09

**Authors:** Sofie Örnö Ax, Michaela Dellenmark-Blom, Kate Abrahamsson, Linus Jönsson, Vladimir Gatzinsky

**Affiliations:** 1 Department of Pediatrics, Institute of Clinical Sciences, The Queen Silvia Children’s Hospital, Gothenburg University, Gothenburg, 416 85 Sweden; 2 Department of Pediatric Surgery, Queen Silvia Children’s Hospital, Sahlgrenska University Hospital, Gothenburg, Sverige

**Keywords:** Esophageal atresia, Feeding difficulties, Eating, Health-related quality of life

## Abstract

**Background:**

Children born with esophageal atresia experience feeding difficulties. This study investigates the association of feeding difficulties and generic health-related quality of life among children aged 2–7 and 8–17 years, born with esophageal atresia.

**Methods:**

108 families (n = 36 aged 2–7 years; n = 72 aged 8–17) answered a survey regarding difficulties in their child’s mealtimes and a validated generic health-related quality of life instrument(PedsQL 4.0). Clinical data was collected from hospital records. The association of feeding difficulties and health-related quality of life was analysed trough Mann-Whitney U-test. Linear regression determined whether the number of concurrent feeding difficulties in the child decreased the health-related quality of life scores. P < 0.05 was considered significant.

**Results:**

In children aged 2–7 and 8–17 years, to have a gastrostomy, to use a food infusion pump, need for energy-enriched food and eating small portions were respectively significantly associated with lower total health-related quality of life scores in the parent-reports (p < 0.05). Most of the feeding difficulties had a negative significant relationship with the domains of physical and social functioning. Additionally, in the older age group, long mealtimes and adult mealtime supervision were associated with lower scores in both child and parent reports. In both age groups, an increased number of feeding difficulties in the child decreased the total generic health-related quality of life scores (p < 0.01).

**Conclusion:**

Specific feeding difficulties are associated with low health-related quality of life among children with esophageal atresia. An increasing number of feeding difficulties is associated to decreasing health-related quality of life-scores. Further research is needed to understand these associations.

**Supplementary Information:**

The online version contains supplementary material available at 10.1186/s13023-023-02836-w.

## Background

Esophageal atresia (EA) is a congenital discontinuation of the esophagus which occurs in one out of every 2,500 to 4,500 live births. Following surgical repair during infancy, the survival rate of EA patients exceeds 90% [1]. About half of the infants born with EA have associated malformations. The most common are congenital heart defects, followed by urinary tract anomalies, gastrointestinal anomalies, and limb defects [2].

Children with repaired EA are reported to experience dysphagia (21–84%), [3–5] gastrointestinal reflux disease (46–76%) [3, 4, 6] and respiratory disorders (21–84%) [6–8]. Long-term medical and psychosocial support is recommended for patients and their families to promote a good health-related quality of life (HRQOL) [4, 9].

“Quality of Life” is defined by the World Health Organization as “the state of complete physical, mental and social well-being and not merely the absence of disease or infirmity” [10] and HRQOL is an adaption of the concept for purposes of health-care practice and research [11, 12]. HRQOL measurements are multidimensional and describe the individual’s experience of the impact of disease and treatment on physical, social and psychological functioning and well-being [13, 14]. The functioning domains have been adapted to measure aspects of HRQOL important to children, and to enable the study of pediatric cohorts [15]. Among patients born with EA, research suggests that symptomatology from the GI tract negatively influences the children’s generic and condition-specific HRQOL, and their family functioning [16–18]. Emotion-focused coping strategies at mealtimes, such as avoidance, distancing or showing fear/worry of nutritional intake are also related to impaired HRQOL [19]. The reported prevalence of eating-related morbidity among EA patients varies in different studies from 7 to 69 per cent [20, 21]. Prevalence of the most frequently reported parent-observed feeding difficulties declines with age throughout childhood [22]. Previous studies of eating-related morbidity among EA patients stresses the importance of early follow-up care by a multidisciplinary team and frequent evaluation with validated assessment instruments. It also suggests parental expectations should be tempered according to type and severity of EA [23–25]. Despite an increase in studies focusing on HRQOL among patients with EA [16], none has focused specifically on how generic HRQOL relates to parent-observed feeding difficulties. This study aims to investigate whether feeding difficulties are associated with a low generic HRQOL and, if so, how they are related to generic HRQOL domains describing levels of social, school, emotional and physical functioning.

## Method

Patients from 2 to 17 years of age, who had had their EA surgically corrected at Queen Silvia Children’s Hospital (QSCH) in Gothenburg, Sweden, and their families, were invited to participate during 2016. The study was conducted as a part of a larger project assessing HRQOL among children born with EA [26]. Following oral and written study information, legal guardians provided written consent for study participation. The study was approved by the Ethical Review Boards of Gothenburg, Sweden (DNR 958 − 13). Demographic and clinical data were collected from a standardized questionnaire and hospital records respectively. At our institution patients attended the Swedish national standardized follow-up program for children with EA, [27] and additionally if clinical problems arose that needed attention.

### Feeding difficulties

A literature review on HRQOL among EA patients identified relevant questions about eating, mealtimes and feeding difficulties that may affect people with EA’s HRQOL [28]. A survey of nine *yes/no-*questions to parents, regarding feeding difficulties caregivers had observed in their child during the past four weeks, was created for use in the study.

### Assessment of HRQOL

HRQOL was measured through the Pediatric Quality of Life Inventory™ 4.0 questionnaire (PedsQL). It was chosen for this study because of its comprehensible design, international use, validation in Swedish population, ability to measure generic HRQOL in pediatric chronic conditions and its domains (physical, social and emotional functioning and functioning at school) which are consistent with the WHO recommendations on measuring HRQOL in children [29–31]. In children with EA, the parent-proxy-report in PedsQL has been confirmed as a reliable source of information of the child’s HRQOL [32]. Items in PedsQL are reverse-scored and linearly transformed to a scale of 0 to 100. A high score indicates good HRQOL. The age-appropriate proxy report for children from 2 to 17 years, and additional age-appropriate child reports for ages from 8 to 17 were sent by post to the families for reply. The questionnaires were returned to the researcher using a pre-stamped envelope.

### Data analysis

Statistical analysis was performed using SPSS 24.0 for Windows (SPSS Inc., Chicago, Illinois, United States). To homogenize the population regarding eating abilities, children with known genetic disorders were excluded, as these are associated with increased feeding and swallowing dysfunction [33]. Since age is identified as being associated with the prevalence of feeding difficulties [22] the population was divided into two age groups, children from 2 to 7 years (preschool or very early school-age), and children from 8 to 17 years (school age). For the two groups, mean values and standard deviations were calculated for continuous variables of the PedsQL 4.0 total and domain function scores. Frequencies and percentages were calculated for categorical values. To determine the association between a feeding difficulty and PedsQL 4.0 scores, the Mann-Whitney U-test was used. A linear regression analysis with the PedsQL 4 − 0 scores as the dependent variable, determined if, and to what extent, the number of feeding difficulties in children with EA decreased the generic HRQOL scores. A score of P < 0.05 was considered significant.

## Results

### Study population

Out of 145 eligible families, 137(95%) accepted the invitation to participate and returned written informed consent. Fifteen families were excluded due to incompletely answered questionnaires, fourteen were excluded as the patients had genetic syndromes and in the older age-group three child reports were excluded due to incomplete data. Population and demographic data, and frequency of feeding difficulties, are shown in Table 1.

**Table 1:** Presentation of study sample: children with esophageal atresia (2–17 years) and prevalence of feeding difficulties

	Children 2–7 years, n = 36	Children 8–17 years, n = 72
	n(%)	n(%)
**Prenatal, congenital factors and variables related to the hospital stay when EA was surgically corrected**		
Prenatal diagnosis of EA	2(6)	8(11)
Male gender	25(69)	39(54)
Prematurity (< 37 weeks)	17(47)	22(31)
Low birth weight (< 2500 gram)	18(50)	24(33)
Associated anomalies	20(56)	40(56)
VACTERL	5(14)	10(14)
Gross type	A:0 B:0 C:33(92) D:2(6) E:1(3)	A:8(11) B:5(7) C:55(76) D:0 E:4(6)
Primary repair of EA	34(95)	60(83)
Revisionary surgery of anastomosis	4(11)	13(18)
Sepsis verified by blood culture	6(17)	10(14)
Esophageal dilatation	12(33)	33(46)
Anastomotic leakage	2(6)	12(17)
**Prevalence of feeding difficulties**		
Avoids specific food due to difficulties eating or swallowing	14 (39)	14 (19)
Eats small portions of food	12 (33)	9 (13)
Supplementary nutrition to increase energy content of diet	8 (22)	9 (13)
Texture modified diet to facilitate eating	12 (33)	2 (3)
More than 30 min to finish main meals*	11 (31)	13 (18)
Needs a lot of fluid with the meal to facilitate swallowing	20 (56)	34 (47)
Gastrostomy	3 (8)	5 (7)
Nutritional intake via a food infusion pump	3 (8)	3 (4)
Eats with extra support from an adult**	9 (25)	5 (7)

* breakfast, lunch, dinner

** example: parent, assistant teacher

### Relationship between feeding difficulties and generic HRQOL

Following an association analysis, lower generic HRQOL scores were associated with several separate feeding difficulties. Feeding difficulties which were significantly associated with lower total scores in the PedsQL parent reports for both the younger (2–7 years) and older (8–17 years) groups were, “supplementary nutrition to increase energy content of diet”, “eats small portions of food*”*, “use of a gastrostomy tube*”* and *“*nutritional intake via a food infusion pump*”* (Table 2). We also found low generic HRQOL scores in both child and parent reports in age group 8 to 17 years concerning the feeding difficulties “more than 30 minutes to finish main meals,” and “eats with extra support from an adult.*”* Among the feeding difficulties associated with low total generic HRQOL scores in both age groups, the physical and social functioning domains were also significantly lower. The same applied for the school domain in the older group. Low HRQOL scores in the emotional domain were significantly associated with only three out of nine feeding difficulties.

**Table 2:** Significant associations of feeding difficulties and health-related quality of life scores (total and functioning domains) according to PedsQL™4.0, in children 2–17 years with repaired esophageal atresia

				PedsQL™4.0 Total score	Total score	Physical functioning domain	Emotional functioning domain	Social functioning domain	School functioning domain
Feeding difficulty				Mean(SD)	p-value	Mean(SD)	Mean(SD)	Mean(SD)	Mean(SD)
Eats small portions of food to facilitate eating		Parent report 2–7 years	NO	90.3(8.1)		93.8(8.1)	85.8(14.3)	95.8(6.5)	80.9(17.0)
			YES	76.7(17.9)	< 0.01	80.7(18.1)**	73.3(19.3)	80.8(19.6)**	68.2(23.0)
		Parent report 8–17 years	NO	86.9(14.5)		88.1(17.4)	84.7(18.3)	90.6(14.7)	83.5(17.3)
			YES	65.8(22.9)	0.01	68.1(21.1)**	68.8(30.6)	65.2(30.3)**	60.4(22.5)*
Supplementary nutrition to increase energy content of diet		Parent report 2–7 years	NO	89.6(9.5)		92.9(8.9)	85.7(14.3)	94.1(9.8)	81.6(17.4)
			YES	72.6(18.1)	< 0.01	77.3(20.0)**	67.5(18.9)**	79.3(20.9)	59.4(18.6)**
		Parent- report 8–17 years	NO	86.7(15.4)		88.1(16.9)	84.2(18.9)	90.5(16.3)	82.9(18.4)
			YES	67.6(20.2)	< 0.01	68.1(23.6)**	72.5(28.9)	65.9(24.0)**	64.9(20.2)*
Gastrostomy tube		Parent-report 2–7 years	NO	90.6(13.5)		90.6(13.5)	83.2(16.8)	92.7(12.8)	78.2(19.9)
			YES	76.0(4.8)	0.02	76.0(4.8)*	65.0(8.7)	70.0(13.2)**	60.0(10.0)
		Parent report 8–17 years	NO	86.2(15.6)		88.0(16.8)	84.2(18.7)	89.8(17.5)	82.0(19.2)
			YES	58.5(16.4)	0.01	53.8(18.4)**	65.0(34.1)	56.0(9.6)**	62.0(11.5)*
Nutritional intake via a food infusion pump		Parent report 2–7 years	NO	87.3(13.1)		90.6(13.5)	83.2(16.8)	92.7(12.8)	78.2(19.9)
			YES	68.8(7.6)	0.01	76.0(4.8)*	65.0(8.7)	70.0(13.2)**	60.0(10.0)
		Parent report 8–17 years	NO	85.1(16.8)		86.9(17.9)	82.9(20.8)	88.7(18.3)	81.1(19.6)
			YES	64.8(12.4)	0.03	56.3(20.5)*	81.7(7.6)	58.3(12.6)**	68.3(7.6)
More than 30 min to finish main meals		Parent report 8–17 years	NO	88.8(12.9)		90.5(13.8)	85.8(18.7)	92.3(13.3)	85.5(15.9)
			YES	63.8(19.2)	< 0.01	63.5(23.4)**	68.3(22.8)*	65.5(25.8)**	58.4(18.9)**
		Child report 8–17 years	NO	88.9(12.7)		90.7(12.5)	87.2(17.8)	92.1(14.5)	84.3(15.6)
			YES	76.4(15.4)	< 0.01	78.4(20.5)	79.5(16.7)	83.5(9.7)*	66.8(14.4)**
Eats with extra support from an adult		Parent report 8–17 years	NO	86.6(14.3)		88.5(15.1)	85.0(17.8)	89.7(16.5)	82.5(17.8)
			YES	52.9(22.4)	0.02	46.9(23.7)*	55.0(32.6)**	57.8(28.0)**	55.0(24.2)*
		Child-report 8–17 years	NO	88.1(12.5)		90.1(13.2)	87.4(15.9)	91.9(13.2)	82.6(16.2)
			YES	59.8(15.2)	0.01	60.4(18.0)**	53.3(28.9)*	66.7(15.3)**	58.3(5.8)*

*p < 0.05

**p < 0.01

As shown in Table 3, linear regression analysis revealed that more parent-reported feeding difficulties were associated with lower total generic HRQOL scores and lower functioning domain scores. The association was significant for all but the school functioning domain for 2–7 year old children. In children aged 2–7 years, a higher number of feeding difficulties decreased the total generic HRQOL scores (p < 0.01, *R*^*2*^ = 25%). In children aged 8–17 years, a higher number of feeding difficulties decreased the total generic HRQOL scores in parent-report (p < 0.01, *R*^*2*^ = 42%) and in child-report (p < 0.01, *R*^*2*^ = 17%).

**Table 3:** Regression analysis of the bearing of nine feeding difficulties* on PedsQL™4.0 scores in children 2–17 years with repaired esophageal atresia

	Physical functioning	Emotional functioning	Social functioning	School functioning	Total PedsQL score
**Children 2–7 years (parent report)**					
β0	96	89	98	83	**93**
β1	-2.7	-2.8	-2.7	-2.5	**-2.7**
R^2^	25%	17%	24%	10%	**25%**
p-value	0.02	0.01	< 0.01	0.06	**< 0.01**
**Children 8–17 years (parent report)**					
β0	96	90	99	89	**94**
β1	-7.6	-5.8	-8.6	-7.4	**-7.3**
R^2^	38%	16%	46%	33%	**42%**
p-value	< 0.01	< 0.01	< 0.01	< 0.01	**< 0.01**
**Children 8–17 years (child report)**					
β0	93	90	94	87	**92**
β1	-3.7	-3.5	-2.9	-4.7	**-3.9**
R^2^	37%	8%	8%	17%	**17%**
p-value	< 0.01	0.02	0.02	< 0.01	**< 0.01**

*listed in Table 1

β0 = constant of each summary scores, e.g. the score in a child without a feeding difficulty

β^1^ = estimate of variation of score, e.g. how scores vary per feeding difficulty present in the child R^2^ = the percentage of the variance in the dependent variable that the independent variables explain collectively (0-100%)

## Discussion

We found that several parent-reported feeding difficulties in children aged from 2 to 7 and from 8 to 17 years were associated with lower generic HRQOL, and that an increasing number of feeding difficulties influenced generic HRQOL negatively.

The presence of four feeding difficulties in children from 2 to 7 and 8–17 years (“eats small portions of food to facilitate eating”, “supplementary nutrition to increase energy content of diet”, “using a gastrostomy tube” and “nutritional intake via a food infusion pump”), were associated with significantly lower total generic HRQOL scores in the parental report. However, the association was not significant in the child report. It has previously been shown that these four feeding difficulties are independently predicted by surgical and/or congenital factors in children with EA [22]. To “take more than 30 minutes to finish a main meal,” and “eat with extra support from an adult,” were significantly associated with low HRQOL in the child and parent reports in the older age group (8 to 17).

The six feeding difficulties listed above were associated with lower scores in the physical functioning domain of the parental report for both age groups. Amin et al. previously observed that the need for long-term follow up for nutritional support of children with EA is associated with lower physical functioning scores [34] and Legrand et al. observed an association between lower scores in the physical functioning domain and gastroesophageal reflux disease [35]. The physical functioning domain of PedsQL 4.0 revolves around the child’s ability to perform daily physical activities (playing, running, managing personal hygiene) and physical discomfort. Our results could indicate that some feeding difficulties captures a subgroup of EA patients with a more severe condition, which is interesting and warrants further research.

We found that the emotional functioning domain was the least impacted by the feeding difficulties mentioned above. This could perhaps be explained by the feeding difficulties assessed in this study, some of which may also be viewed as behaviour/actions taken by the child to try and eliminate dysphagia or choking (for example eating slowly or eating small portions). In fact, some of these actions could fit the description of problem-solving focused coping strategies in nutritional intake situations, [26] which are associated with good HRQOL [36]. Dysphagia has been shown to be associated with lower emotional functioning scores in PedsQL [35]. The weak association with the emotional functioning indicates that parent-observed items in this study might not be enough to capture children’s subjective experiences of dysphagia.

To “take more than 30 minutes to finish main meals,” and “eat with extra support from an adult,” were associated with lower total HRQOL for both children and their parents in the older group. A hypothesis regarding these results is that children aged 8–17 years might be bothered by feeding difficulties that affect social interactions with their peers and family during mealtimes. This is supported by the association of these two feeding difficulties with low scores in the social and school functioning domains in child and parent reports. The social functioning domain of PedsQL asks about the child’s ability to make friends, social exclusion, and his/her ability to agree with and socialize with peers. The school functioning domain focuses on the child’s ability to keep up with schoolwork, to concentrate in school and whether he/she misses out on school due to health issues. Previous studies have shown that when using a condition-specific approach in the older EA group, both social aspects of eating and social isolation are prominent HRQOL issues from both a child and parent’s perspective.[26] All but one (“supplementary nutrition to increase energy content of diet”) of the six feeding difficulties associated with low HRQOL were associated with low scores in the social functioning domain.

Our study showed that for children with EA, an increase in the number of feeding difficulties is associated with a decrease in generic HRQOL. The cumulative negative relationship is important for young children (2 to 7 years) with EA, who commonly have one or several feeding difficulties (> 60%) [22]. The linear association between the number of feeding difficulties and HRQOL implies that feeding difficulties are an important influence on generic HRQOL among children with EA. Since only 25% of the negative association between feeding difficulties and the 2–7 year old child’s lower generic HRQOL could be explained by our model, there are evidently other factors along with parent-reported feeding difficulties that impact HRQOL. The explanation rate for the 8-17year-olds was higher in the parental report (42%) and lower in the child report (17%). Other than feeding difficulties, concurrent anomalies, GERD, barky cough and prematurity have been associated with lower generic HRQOL in children with EA [37, 38]. When taking a condition-specific approach, HRQOL is impaired by several congenital and surgical factors, but throughout childhood it is digestive symptoms which impact EA-specific HRQOL the most [18]. Some of the parent-reported feeding difficulties might be connected to specific EA-related morbidities which in their turn cause low HRQOL. Further research is warranted to determine how feeding difficulties relate to other EA-related symptomatology.

The relationship between feeding difficulties and generic HRQOL in our study was analyzed through a widely used validated generic HRQOL instrument. The nine questions assessing feeding difficulties may not fully comprise EA-specific eating morbidity and some of the feeding difficulties (such as to have a gastrostomy and nutritional intake via food infusion pump) may be interrelated. Still, of importance, our results indicate that these feeding difficulties have impact on HRQOL. The prevalence and character of feeding difficulties may differ in different child ages. The age range of the groups was chosen because of the cut-off ages in the PedsQL-instrument (self-report from age 8) and as Swedish children start school at age seven. Therefore the patient material was divided into two age groups. The older group (8 to 17 years) was twice as big as the younger (2 to 7 years) group due to its wider age range. A further division of subgroups was not carried out because even as our material comes from one of the largest cohorts in the field, [16] the low prevalence of some feeding difficulties would make statistical analysis less feasible. In order to further understand the relationship between feeding difficulties and HRQOL, including and how to target treatment and follow up, a standardized feeding difficulty score for children with EA should be developed to serve in a multicenter study and enable assessment of a multifactorial model of HRQOL, identifying the specific contribution of feeding difficulties on HRQOL scores in children with EA.

## Conclusions

The presence of feeding difficulties is associated with low generic HRQOL among children with EA, and areas of physical and social functioning are especially affected. For children aged 8 to 17 years, feeding difficulties that impacts social interactions with their peers and family during mealtimes are associated with low generic HRQOL. An increasing number of feeding difficulties is associated with lower generic HRQOL, which is important to recognize during early follow-ups when the prevalence of feeding difficulties is high.

### Electronic supplementary material

Below is the link to the electronic supplementary material.

Supplementary Material 1

## Data Availability

The datasets analyzed during the current study are available in the manuscript. Further information in earlier publication or is not available in public due to lack of ethical approval.
