# Supplementary material for: The association of feeding difficulties and generic health-related quality of life among children born with esophageal atresia
Source: Orphanet J Rare Dis. 2023 Aug 9;18:237. doi: 10.1186/s13023-023-02836-w (PMC10410866; doi:10.1186/s13023-023-02836-w)
Supplement: Supplementary file 1 — Supplementary Material 1 [file 13023_2023_2836_MOESM1_ESM.docx]

| **Supplemental Table 1: Description of health-related quality of life according to PedsQL™4.0 scores in children with repaired esophageal atresia aged 2-7 years (n=36), reported to have or not to have feeding difficulties (parental report)** | | | | | | | | | | | | | | | | |  |  |
| --- | --- | --- | --- | --- | --- | --- | --- | --- | --- | --- | --- | --- | --- | --- | --- | --- | --- | --- |
|  |  |  | PHYSICAL FUNCTIONING | | | EMOTIONAL FUNCTIONING | | | SOCIAL FUNCTIONING + | | | SCHOOL FUNCTIONING | | | TOTAL HRQOL± | | | |
|  | NO | YES | NO Mean(SD) | YES Mean(SD) | p-value | NO Mean(SD) | YES Mean(SD) | p-value | NO Mean(SD) | YES Mean(SD) | p-value | NO Mean(SD) | YES Mean(SD) | p-value | NO Mean(SD) | YES Mean(SD) | | p-value |
| The child avoids food that is difficult to eat or swallow | 22 | 14 | 92,2(8,8) | 85,0(18,4) | 0,23 | 83,4(16,4) | 78,9(18,1) | 0,48 | 93,0(11,2) | 87,5(17,8) | 0,38 | 77,9(18,6) | 74,8(22,1) | 0,70 | 87,8(10,7) | 82,6(17,3) | | 0,40 |
| The child is eating smaller portions so that it is easier to eat | 24 | 12 | 93,8(8,1) | 80,7(18,1) | 0,01** | 85,8(14,3) | 73,3(19,3) | 0,04* | 95,8(6,5) | 80,8(19,6) | 0,01** | 80,9(17,0) | 68,2(23,0) | 0,11 | 90,3(8,1) | 76,7(17,9) | | 0,01** |
| The child needs energy enriched  food | 28 | 8 | 92,9(8,9) | 77,3(20,0) | 0,01** | 85,7(14,3) | 67,5(18,9) | 0,01** | 94,1(9,8) | 79,3(20,9) | 0,07 | 81,6(17,4) | 59,4(18,6) | 0,01** | 89,6(9,5) | 72,6(18,1) | | 0,01** |
| The child needs texture modified meals to facilitate eating | 24 | 12 | 92,4(8,4) | 83,3(19,4) | 0,19 | 83,5(15,8) | 77,9(19,2) | 0,41 | 93,1(10,8) | 86,3(19,0) | 0,33 | 79,5(18,6) | 71,0(21,7) | 0,24 | 88,3(10,4) | 80,8(18,1) | | 0,21 |
| The child takes a long time (>30 minutes) to finish a main meal | 25 | 11 | 92,0(9,5) | 83,5(19,4) | 0,09 | 84,8(14,4) | 74,5(20,8) | 0,15 | 92,6(11,0) | 86,8(19,7) | 0,42 | 77,7(19,4) | 74,2(21,5) | 0,58 | 88,1(10,4) | 80,4(18,7) | | 0,29 |
| The child needs increased fluid intake during meals to facilitate swallowing | 16 | 20 | 93,2(9,0) | 86,4(15,9) | 0,11 | 85,9(11,4) | 78,3(20,0) | 0,32 | 95,3(8,1) | 87,3(17,0) | 0,07 | 80,0(19,0) | 74,0(20,5) | 0,40 | 89,9(9,3) | 82,5(15,8) | | 0,18 |
| The child needs to be fed through a gastrostomy | 33 | 3 | 90,6(13,5) | 76,0(4,8) | 0,02* | 83,2(16,8) | 65,0(8,7) | 0,05 | 92,7(12,8) | 70,0(13,2) | 0,01** | 78,2(19,9) | 60,0(10,0) | 0,09 | 87,3(13,1) | 68,8(7,6) | | 0,01* |
| The child needs to be fed through an infusion pump | 33 | 3 | 90,6(13,5) | 76,0(4,8) | 0,02* | 83,2(16,8) | 65,0(8,7) | 0,05 | 92,7(12,8) | 70,0(13,2) | 0,01** | 78,2(19,9) | 60,0(10,0) | 0,09 | 87,3(13,1) | 68,8(7,6) | | 0,01* |
| The child needs support by an adult during meals | 27 | 9 | 91,5(9,0) | 83,0(21,9) | 0,37 | 84,4(14,6) | 73,3(21,5) | 0,17 | 93,0(10,7) | 84,4(21,1) | 0,26 | 78,2(18,8) | 72,3(23,2) | 0,38 | 88,0(10,0) | 79,1(20,6) | | 0,28 |
| + 1 missing  ± Health-related quality of life  *p<0,05  **p<0,01 | | | | | | | | | | | | | | | | | | |

| **Supplemental Table 2: Description of health-related quality of life according to PedsQL™4.0 scores in children with repaired esophageal atresia aged 8-17 years (n=69), reported to have or not to have feeding difficulties (child report)** | | | | | | | | | | | | | | | | |  |  |
| --- | --- | --- | --- | --- | --- | --- | --- | --- | --- | --- | --- | --- | --- | --- | --- | --- | --- | --- |
|  |  |  | PHYSICAL FUNCTIONING | | | EMOTIONAL FUNCTIONING | | | SOCIAL FUNCTIONING + | | | SCHOOL FUNCTIONING | | | TOTAL HRQOL± | | | |
|  | NO | YES | NO  Mean(SD) | YES  Mean(SD) | p-value | NO  Mean(SD) | YES  Mean(SD) | p-value | NO  Mean(SD) | YES  Mean(SD) | p-value | NO  Mean(SD) | YES  Mean(SD) | p-value | NO  Mean(SD) | YES  Mean(SD) | | p-value |
| The child avoids food that is difficult to eat or swallow | 56 | 13 | 89,1(14,8) | 87,5(14,3) | 0,43 | 86,8(17,1) | 82,3(20,8) | 0,69 | 90,4(15,3) | 92,9(7,5) | 0,85 | 81,6(16,9) | 81,2(16,1) | 0,88 | 87,2(13,9) | 85,3(13.7) | | 0,42 |
| The child is eating smaller portions so that it is easier to eat | 62 | 7 | 89,8(13,6) | 79,5(20,9) | 0,18 | 89,8(17,8) | 81,4(17,7) | 0,35 | 91,1(14,3) | 87.5(13,7) | 0,34 | 83,5(15,7) | 63,6(13,8) | 0,01** | 88,0(13,1) | 76,7(17,1) | | 0,06 |
| The child needs energy enriched  food | 60 | 9 | 89,9(12,7) | 81,3(23,7) | 0,65 | 87,2(15,9) | 77,8(26,9) | 0,36 | 92,3(11,0) | 79,4(27,2) | 0,12 | 83,2(15,6) | 70,6(20,1) | 0,07 | 88,4(11,7) | 76,8(21,8) | | 0,21 |
| The child needs texture modified meals to facilitate eating | 67 | 2 | 89,2(14,0) | 75,0(35,4) | 0,58 | 86,5(17,7) | 67,5(10,6) | 0,10 | 90,8(14,3) | 90 (19,5) | 0,53 | 82,0(16,4) | 65,0(21,2) | 0,14 | 87,4(13,3) | 69,0(26,9) | | 0,12 |
| The child takes a long time (>30 minutes) to finish a main meal | 58 | 11 | 90,7(12,5) | 78,4(20,5) | 0,05 | 87,2(17,8) | 79,5(16,7) | 0,09 | 92,1(14,5) | 83,5(9,7) | 0,001* | 84,3(15,6) | 66,8(14,4) | 0,01** | 88,9(12,7) | 76,4(15,4) | | 0,01** |
| The child needs increased fluid intake during meals to facilitate swallowing | 39 | 30 | 91,2(12,9) | 85,5(16,3) | 0,12 | 88,1(17,9) | 83,2(17,5) | 0,20 | 91,0(16,6) | 90,5(10,4) | 0,17 | 84,5(16,0) | 77,7(16,9) | 0,07 | 89,0(13,4) | 84,1(14,0) | | 0,07 |
| The child needs to be fed through a gastrostomy | 63 | 6 | 89,7(13,8) | 79,4(20,6) | 0,20 | 87,1(15,8) | 73,3(31,6) | 0,33 | 92,6(10,8) | 72,5(28,9) | 0,03* | 82,5(16,6) | 70,8(14,3) | 0,07 | 88,0(12,5) | 86,9(13,8) | | 0,07 |
| The child needs to be fed through an infusion pump | 65 | 4 | 88,9(14,5) | 87,1(18,5) | 0,92 | 85,8(17,8) | 88,8(19,3) | 0,71 | 90,9(14,5) | 90,0(8,2) | 0,41 | 81,8(16,9) | 77,5(12,6) | 0,39 | 86,9(13,9) | 86,0(14,3) | | 0,62 |
| The child needs support by an adult during meals | 66 | 3 | 90,1(13,2) | 60,4(18,0) | 0,01** | 87,4(15,9) | 53,3(28,9) | 0,02* | 91,9(13,2) | 66,7(15,3) | 0,01** | 82,6(16,2) | 58,3(5,8) | 0,03* | 88,1(12,5) | 59,8(15,2) | | 0,01* |
| + 1 missing  ± Health-related quality of life  *p<0,05  **p<0,01 | | | | | | | | | | | | | | | | | | |

| **Supplemental Table 3: Description of health-related quality of life according to PedsQL™4.0 scores in children with repaired esophageal atresia aged 8-17 years (n=72), reported to have or not to have feeding difficulties (parental report)** | | | | | | | | | | | | | | | | |  |
| --- | --- | --- | --- | --- | --- | --- | --- | --- | --- | --- | --- | --- | --- | --- | --- | --- | --- |
|  |  |  | PHYSICAL FUNCTIONING | | | EMOTIONAL FUNCTIONING + | | | SOCIAL FUNCTIONING + | | | SCHOOL FUNCTIONING | | | TOTAL HRQOL± | | |
|  | NO | YES | NO Mean(SD) | YES Mean(SD) | p-value | NO Mean(SD) | YES Mean(SD) | p-value | NO Mean(SD) | YES Mean(SD) | p-value | NO Mean(SD) | YES Mean(SD) | p-value | NO Mean(SD) | YES Mean(SD) | p-value |
| The child avoids food that is difficult to eat or swallow (+) | 57 | 14 | 87,6(17,2) | 79,7(23,6) | 0,28 | 84,5(20,4) | 75,0(19,5) | 0,05 | 89,7(16,4) | 78,4(26,8) | 0,05 | 82,0(19,1) | 73,8(20,5) | 0,14 | 86,2(16,1) | 76,9(20,0) | 0,08 |
| The child is eating smaller portions so that it is easier to eat | 63 | 9 | 88,1(17,4) | 68,1(21,1) | 0,01** | 84,7(18,3) | 68,8(30,6) | 0,14 | 90,6(14,7) | 65,2(30,3) | 0,01** | 83,5(17,3) | 60,4(22,5) | 0,01* | 86,9(14,5) | 65,8(22,9) | 0,01* |
| The child needs energy enriched  food | 63 | 9 | 88,1(16,9) | 68,1(23,6) | 0,01** | 84,2(18,9) | 72,5(28,9) | 0,14 | 90,5(16,3) | 65,9(24,0) | 0,01** | 82,9(18,4) | 64,9(20,2) | 0,02* | 86,7(15,4) | 67,6(20,2) | 0,01** |
| The child needs texture modified meals to facilitate eating | 70 | 2 | 86,0(19,0) | 75,0(17,7) | 0,20 | 82,9(20,5) | 80,0(???) | 0,60 | 88,3(18,2) | 56,7(33,0) | 0,05 | 81,2(19,2) | 59,4(22,1) | 0,16 | 84,8(16,9) | 66,6(21,1) | 0,13 |
| The child takes a long time (>30 minutes) to finish a main meal | 59 | 13 | 90,5(13,8) | 63,5(23,4) | 0,01** | 85,8(18,7) | 68,3(22,8) | 0,01* | 92,3(13,3) | 65,5(25,8) | 0,01** | 85,5(15,9) | 58,4(18,9) | 0,01** | 88,8(12,9) | 63,8(19,2) | 0,01** |
| The child needs increased fluid intake during meals to facilitate swallowing | 38 | 34 | 88,8(18,1) | 82,0(19,4) | 0,03* | 84,7(19,5) | 80,8(21,5) | 0,44 | 90,8(15,7) | 83,7(15,7) | 0,07 | 84,5(17,9) | 76,3(20,4) | 0,05 | 87,4(15,7) | 80,8(18,1) | 0,05 |
| The child needs to be fed through a gastrostomy | 67 | 5 | 88,0(16,8) | 53,8(18,4) | 0,01** | 84,2(18,7) | 65,0(34,1) | 0,07 | 89,8(17,5) | 56,0(9,6) | 0,01** | 82,0(19,2) | 62,0(11,5) | 0,02* | 86,2(15,6) | 58,5(16,4) | 0,01* |
| The child needs to be fed through an infusion pump | 69 | 3 | 86,9(17,9) | 56,3(20,5) | 0,02* | 82,9(20,8) | 81,7(7,6) | 0,44 | 88,7(18,3) | 58,3(12,6) | 0,01** | 81,1(19,6) | 68,3(7,6) | 0,15 | 85,1(16,8) | 64,8(12,4) | 0,03* |
| The child needs support by an adult during meals | 67 | 5 | 88,5(15,1) | 46,9(23,7) | 0,01* | 85,0(17,8) | 55,0(32,6) | 0,01** | 89,7(16,5) | 57,8(28,0) | 0,01** | 82,5(17,8) | 55,0(24,2) | 0,01* | 86,6(14,3) | 52,9(22,4) | 0,02* |
| + 1 missing  ± Health-related quality of life  *p<0,05  **p<0,01 | | | | | | | | | | | | | | | | | |
